# Worldwide productivity in the field of foot and ankle research from 2009–2013: a bibliometric analysis of highly cited journals

**DOI:** 10.1186/s13047-015-0070-0

**Published:** 2015-04-14

**Authors:** Xuyao Luo, Zhimin Liang, Feng Gong, Hongwei Bao, Li Huang, Zhiwei Jia

**Affiliations:** Department of Orthopaedics, Hospital 411 of CPLA, Shanghai, China; Graduate School, Second Military Medical University, NO.800 Xiangyin Road, 200433 Shanghai, China

**Keywords:** Foot and ankle research, Bibliometric analysis

## Abstract

**Background:**

Significant growth has been observed in the field of foot and ankle research in recent years. However, bibliometric studies concerning the quantity and quality of articles published in foot and ankle journals are scarce. This study aimed to reveal the characteristics of national productivity in the field of foot and ankle research and to provide a general picture of foot and ankle research for surgeons and researchers.

**Methods:**

Web of Science was searched for foot and ankle articles in 4 highly cited journals from 2009 to 2013. The number of total articles and citations were collected to evaluate the contribution of different countries. Publication activity was adjusted for the countries by population size and gross domestic product (GDP).

**Results:**

A total number of 2083 articles were published worldwide. North America, West Europe, Australia and East Asia were the most productive world regions. High income countries published 90.35% of articles, middle-income 9.60%, and low-income just 0.05%. The United States published the largest number of articles (1025/2083, 49.2%), followed by the United Kingdom (221/2083, 10.6%), Australia (92/2083, 4.4%), and had the highest total citations (3631). However, Canada had the highest average citations per article (5.0), followed by Australia (4.6) and Switzerland (4.2). There were positive correlations between the total number of publications and population/GDP (*p* < 0.01). When normalized to population size, Switzerland ranked the highest, followed by Australia, and the United Kingdom. When adjusted for GDP, Switzerland ranked the highest, followed by United Kingdom, and South Korea.

**Conclusions:**

The United States is the most productive country in the field of foot and ankle research. However, Australia, some smaller European and Asian countries may be more productive relative to their size.

## Background

In recent years, significant progress has been seen in the field of foot and ankle research. Worldwide contributions are responsible for this dramatic growth. However, the scientific contribution to the field of foot and ankle research is unlikely to be equal for each country, since different countries have different healthcare systems, financial research sources and scientific research programs [1,2].

Publication, as a central part of the scientific research process, is important for the advancement of the field of foot and ankle research. The number of articles published by a country is an indicator of its contributions to the creation of new knowledge, and bibliometric analysis is often used to investigate trends in scholarly publications and the relative importance of articles in a specific topic. Recently, bibliometric analysis for assessing the worldwide research productivity has been increasingly performed in various medical fields, such as surgical oncology [2], emergency medicine [3], anaesthesia [4], critical care medicine [5], rheumatology [6], and plastic and reconstructive surgery [7].

However, to the best of our knowledge, bibliometric studies concerning the quantity and quality of articles published in the field of foot and ankle research have never been reported before. Therefore the objective of the present study was to investigate the characteristics of national productivity in the field of foot and ankle research and to provide an insight into the status of the world foot and ankle research for surgeons and researchers.

## Methods

The structure of this study was modeled on previous similar publications [3-7]. A total of 4 highly cited journals related to foot and ankle research were selected from the “Orthopedics” category of the 2013 Journal Citation Reports (JCR) (Thomson Reuters, New York, USA) [8]. The 4 journals were listed in Table 1. A computerized literature search was conducted in the database of Web of Science (WoS) (Thomson Reuters, New York, USA) in November 10, 2014. This platform was chosen because it was the world’s leading database collecting citation and other academic impact information, and had been widely used in similar studies [3,5,7]. Only original articles and reviews were included. Letters, editorial material and correction were excluded. Where there was more than one institutional affiliation listed, the source nation was taken as the country of the corresponding author.

**Table 1:** **Journals included in the search**

**Journal**	**Abbreviation**	**Impact factor**
Journal of Foot and Ankle Research	JFAR	1.831
Foot & Ankle International	FAI	1.626
Journal of Foot & Ankle Surgery	JFAS	0.979
Foot and Ankle Clinics	FAC	0.844

The number of published articles was considered as an index of quantity of research productivity. The number of citations was considered as a quality indicator. The primary outcome was the number of articles attributed to each country. To reveal the contribution of different countries, the countries were ranked according to their productivity. Based on the categories of World Bank, we also calculated the proportion of articles that was attributed to high income, upper middle income, lower middle income, and low income countries [9]. This categorization in terms of gross national income (GNI) per capita includes high income, $12746 or more; upper middle income, $4126 to $12745; lower middle income, $1046 to $4125; and low income, $1045 or less [9]. Moreover, the research productivity of different countries was evaluated in relation to population size and gross domestic product (GDP). These data for each country were gathered from the Central Intelligence Agency and Word Bank for the most recent report [9,10].

We further comprehensively analyzed the publications of the main productive countries (producing at least 1% of the total publications), including the total numbers, the per capita numbers adjusted for population and GDP, total citations, and mean citations. Publications in the 4 journals from the top 5 countries were generated, and the top 3 countries in the journals were listed.

Because our goal was to describe trends and not to test hypotheses about the relative contribution of different countries, only simple descriptive statistics (e.g., sum or average) were used. The statistical significance of the correlation was determined by Spearman’s test [6]. All the statistical tests were performed using SPSS software version 19.0 (SPSS Inc., Chicago, IL, USA), and *p* < 0.05 was considered to be statistically significant.

## Results

**Figure 1:**
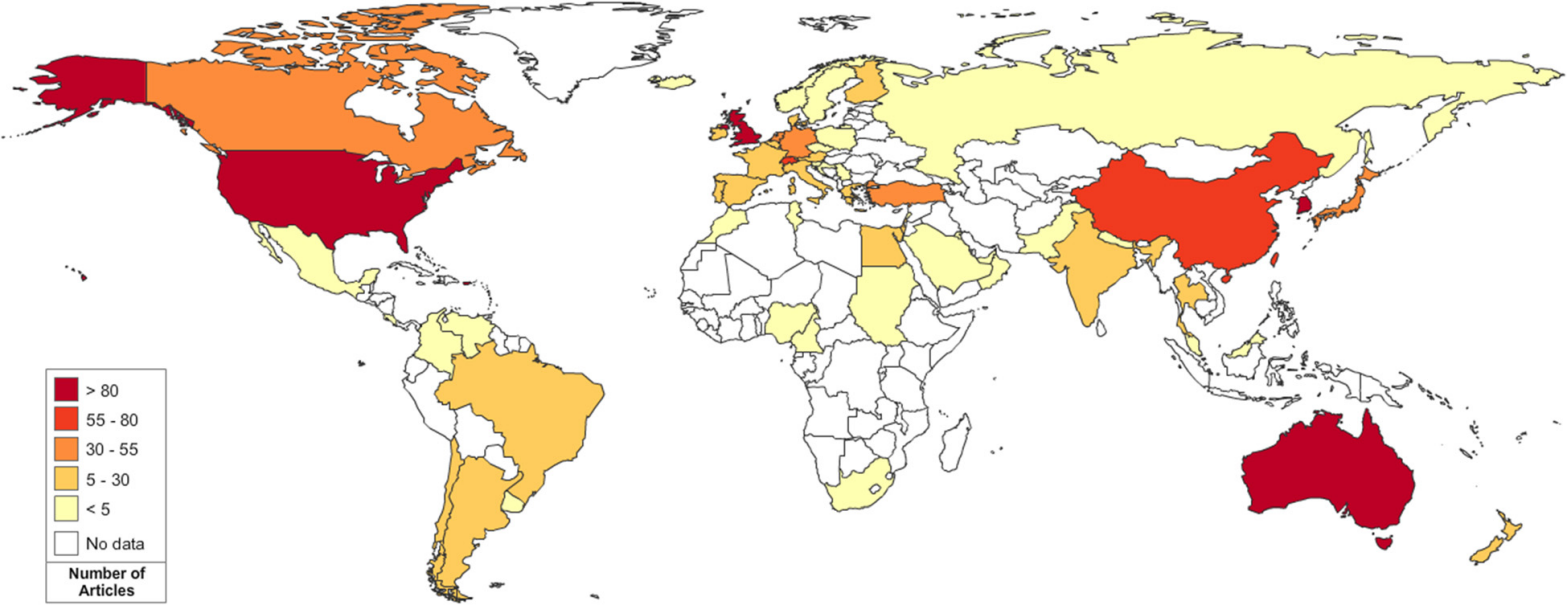
The world map of worldwide research productivity in 2009 to 2013.

**Figure 2:**
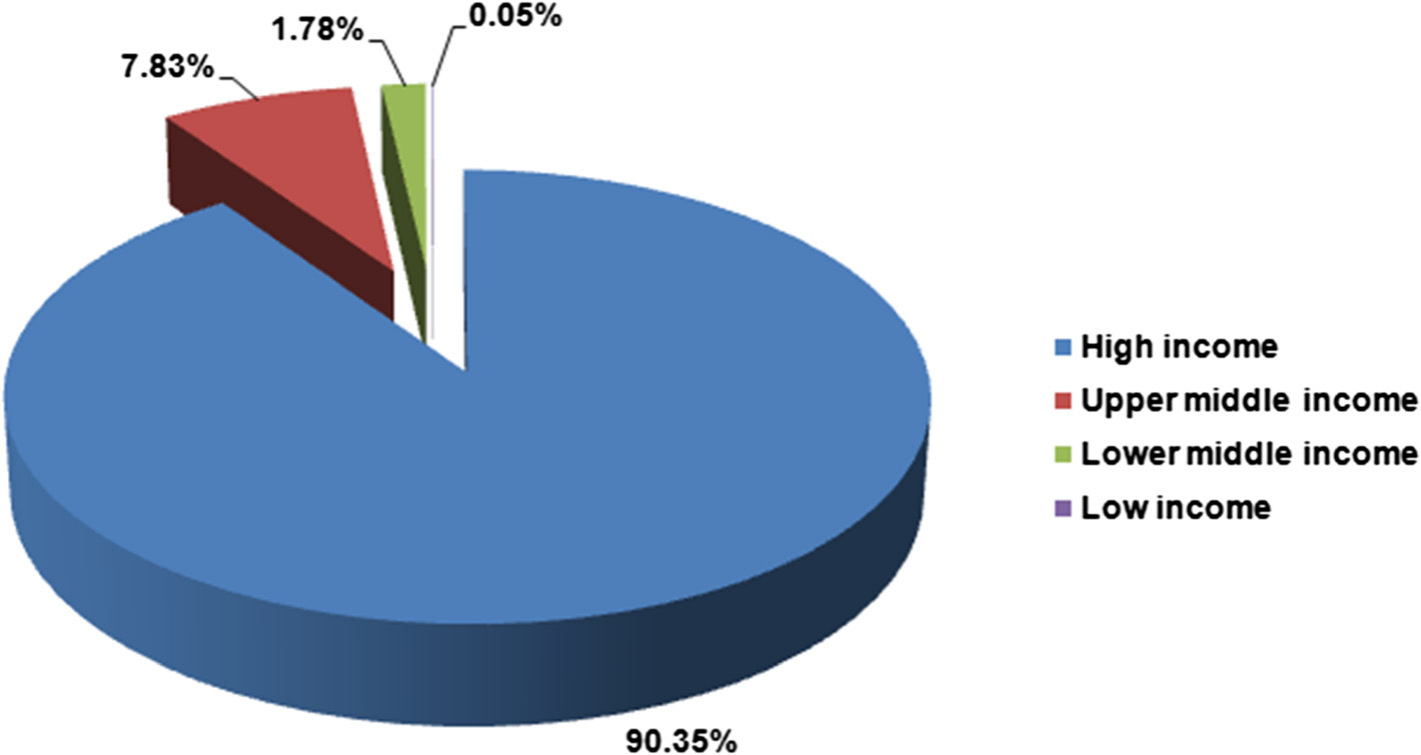
Publications grouped by gross national income in 2009 to 2013.

**Figure 3:**
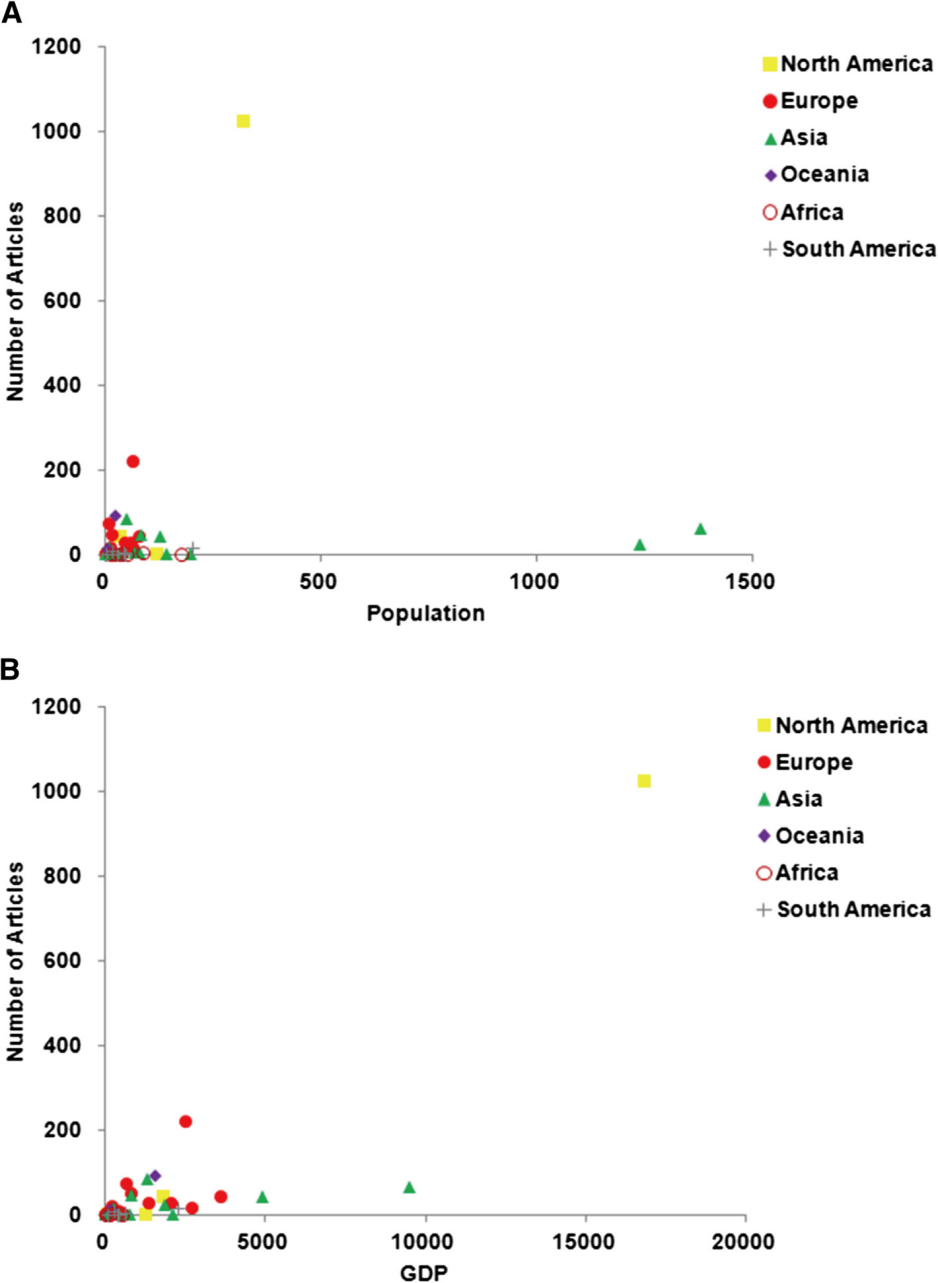
Scatter plot showing the association of publication activity in the field of foot and ankle research with population (**A**) and GDP (**B**) from different countries.

A total of 2083 articles on foot and ankle research were identified in the database of Web of Science from 2009 to 2013. A total of 59 countries contributed to the development of the field of foot and ankle research. The United States published the most number of articles (1025/2083, 49.2%), followed by the United Kingdom (221/2083, 10.6%), and Australia (92/2083, 4.4%). North America was the most productive continent (51.4%), followed by Europe (25.6%), Asia (15.2%), Oceania (5.1%), South America (1.7%), and Africa (0.9%). The world map of worldwide research productivity is shown in Figure 1, indicating that North America, West Europe, Australia and East Asia were the most productive regions from 2009 to 2013. Moreover, high-income countries published 1882 articles (90.35%), and middle-income countries (sum of upper middle-income and lower middle-income countries) published 200 articles (9.60%). However, low-income countries published only 1 article (0.05%) (Figure 2). The numbers of publications showed significant correlations (*p* < 0.01) with population size and GDP (*r* = 0.380 and *r* = 0.646, respectively) (Figure 3).

A total of 14 main productive countries (producing at least 1% of total articles) published 89.8% (1870/2083) of the total articles (Table 2). Most of them were high-income countries (11). The 6th, 8th and 14th ranked nations (China, Turkey and India respectively) were classified as middle income countries. Among the 14 countries, the United States had the highest total citations (3631), followed by the United Kingdom (651), and Australia (420). Canada had the highest mean citations (5.0), followed by Australia (4.6), and Switzerland (4.2).

**Table 2:** **Articles from the most productive countries, 2009-2013**

**Country**	**N**	**%**	**N per 10 million population**	**N per 100 billion US $ GDP**	**Total citation**	**Mean citation**
United States	1025	49.2	32.1	6.1	3631	3.5
United Kingdom	221	10.6	34.7	8.8	651	2.9
Australia	92	4.4	40.9	5.9	420	4.6
South Korea	84	4.0	17.1	6.4	162	1.9
Switzerland	72	3.5	89.3	11.1	300	4.2
China	64	2.4	0.5	0.7	119	1.9
Netherlands	49	2.4	29.0	6.1	147	3.0
Turkey	48	2.3	5.9	5.9	113	2.4
Germany	45	2.2	5.6	1.2	124	2.8
Japan	45	2.2	3.5	0.9	76	1.7
Canada	44	2.1	12.6	2.4	221	5.0
Italy	29	1.4	4.7	1.4	95	3.3
Spain	28	1.3	5.9	2.1	62	2.2
India	24	1.2	0.2	1.3	17	0.7

*Abbreviations: N* number, *GDP* Gross Domestic Product.

Regarding the production per capita, Switzerland had the highest number of articles (89.3), followed by Australia (40.9), and the United Kingdom (34.7). When number of articles adjusted for GDP, Switzerland ranked the highest in the top list (11.1), followed by the United Kingdom (8.8), and South Korea (6.4). Countries with large economies, such as the United States, China and Japan, tended to rank relatively low after adjustment for GDP.

The publications from the top 5 countries are shown in Table 3. Among the top 5 countries, *Foot & Ankle International* (FAI) was the most popular journal in 4 countries, including United States, United Kingdom, South Korea and Switzerland; *Journal of Foot and Ankle Research* (JFAR) was the most popular in Australia.

**Table 3:** **Publications from the top countries**

**Rank**	**United States**	**United Kingdom**	**Australia**	**South Korea**	**Switzerland**
1	FAI (455)	FAI (76)	JFAR (65)	FAI (57)	FAI (40)
2	JFAS (430)	JFAS (59)	FAI (17)	JFAS (21)	FAC (24)
3	FAC (126)	JFAR (58)	JFAS (8)	FAC (5)	JFAS (7)
4	JFAR (14)	FAC (28)	FAC (2)	JFAR (1)	JFAR (1)

*Abbreviations: JFAR* Journal of Foot and Ankle Research, *FAI* Foot & Ankle International, *JFAS* Journal of Foot & Ankle Surgery, *FAC* Foot and Ankle Clinics.

FAI published the largest number of foot and ankle articles (936/2083, or 44.9%), followed by *Journal of Foot & Ankle Surgery* (JFAS) (748/2083, or 35.9%), *Foot and Ankle Clinics* (FAC) (228/2083, or 10.9%), and JFAR (171/2083, or 8.2%). The 3 most productive countries in the 4 journals are listed in Table 4. The United States was the most productive country in 3 journals including FAI, JFAS and FAC, while Australia was the most productive country in JFAR. In addition, the United States and United Kingdom appeared in the top 3 countries in all the 4 journals.

**Table 4:** **Top ranked countries by journal**

**Rank**	**FAI**	**JFAS**	**FAC**	**JFAR**
1	United States (455)	United States (430)	United States (126)	Australia (65)
2	United Kingdom (76)	United Kingdom (59)	United Kingdom (28)	United Kingdom (58)
3	South Korea (57)	Turkey (30)	Switzerland (24)	United States (14)

*Abbreviations: JFAR* Journal of Foot and Ankle Research, *FAI* Foot & Ankle International, *JFAS* Journal of Foot & Ankle Surgery, *FAC* Foot and Ankle Clinics.

## Discussion

Foot and ankle research has recently experienced a considerable evolution, which can be attributed to the contributions by researchers and surgeons from all over the world. To assess the research contributions around the world, biomedical research publication has been used as an index for scientific research productivity [2-7]. Recently, many studies using bibliometric methods have evaluated worldwide research productivity in several biomedical fields [2-7]. However, as far as we know, this study is the first bibliometric evaluation on worldwide productivity in the field of foot and ankle research.

The present study found that the authors originating from the United States published far more articles than any other country. It is no surprise that the United States leads the rankings, which also had been found in many fields of medicine [2-7]. Therefore this result confirms the major influence of the United States in the field of foot and ankle research.

Besides the most number of articles, the United States also had the highest total citation. Most importantly, the United States also had high mean citations, which suggested that publications originating from the United States had not only large quantity but also high quality. Based on these large number of high-quality studies, policy makers and healthcare practitioners could inform successful interventions and may further improve clinical practice [11]. Although the United States has a large population, the per capita numbers of articles from the United States remained one of the most numbers per capita. These results suggest that the United States is the most productive country in the field of foot and ankle research in the world.

Regarding the contributions of different countries, a “10/90” divide was used to described the proportion between non-high and high income countries [12], which had been demonstrated in some medical fields [3-5,7]. It also holds true for the field of foot and ankle research. Only three non-high income countries, including China, Turkey and India, are listed in the main productive countries. The increasing importance of these countries has been shown in previous studies [3,4,13,14]. Moreover, our results demonstrate that GDP is also a positive factor related to research productivity besides population size [6]. The high research productivity can be a suggestive reflection of the rapid development of society and economy in these middle income countries [3,4]. It may be forecasted that these countries with rapid economic development could further improve their foot and ankle research and promote their ranks in the future. The lack of research productivity in low income countries was observed in this study for only three articles identified. This may be affected by a combination of factors, such as government policy, medical infrastructures, research fund and researchers [4,12]. This result indicates the underrepresentation of non-high income countries in publications of foot and ankle research, despite these countries have the largest burden of musculoskeletal disease [11]. In these countries, establishing strategies to increase the number of high-quality researches may improve the evidence-based health policies and patient care [11].

When the total number of articles adjusted by population and GDP, Australia, some European countries such as the United Kingdom and Switzerland, and Asian countries such as South Korea are more productive. Although it may make more sense to normalize by the number of researchers and GDP invested in foot and ankle research in each country, not the population size and total GDP, it is rather difficult to obtain these data in the field of foot and ankle research in each country. Nonetheless, this result demonstrates the high scientific research output of these smaller countries.

The United States was the most productive country in 3 journals, including FAI, JFAS and FAC. It should be recognized that these journals are all published in the United States. More submissions might therefore be from the United States than from other countries. In addition, the United States and the United Kingdom appears in the top 3 countries in all the 4 journals, indicating the important influence of these two countries in the field of foot and ankle research.

Besides the most number of articles in 4 of the top 5 countries, FAI published the largest number of foot and ankle research worldwide, which indicated the importance of FAI in the body of foot and ankle publications [15].

There are some limitations in this study. Although the journals were selected from the orthopedics category of the JCR, some general orthopedics and basic research journals, which also published some articles related to foot and ankle research, were not included in this study. In addition, citations are only one measure of research impact, and may not reflect the influence of each article. Finally, 3 of the 4 journals included in our study (FAI, JFAS and FAC) have a strong surgical focus, so our findings may not translate to research into conservative interventions for foot and ankle disorders.

## Conclusion

This is the first bibliometric study assessing the worldwide productivity in the field of foot and ankle research. This study demonstrates that the United States is the most productive country in the field of foot and ankle research. However, Australia, some smaller European and Asian countries might be more productive relative to their population size and GDP.

## Abbreviations

GDP: Gross domestic product
JCR: Journal Citation Reports
WoS: Web of Science
FAI: Foot & Ankle International
JFAR: Journal of Foot and Ankle Research
JFAS: Journal of Foot & Ankle Surgery
FAC: Foot and Ankle Clinics
